# Prevalence and factors associated with intention to use contraceptives among women of reproductive age: a multilevel analysis of the 2018 Guinea demographic and health survey

**DOI:** 10.1186/s12884-023-06204-1

**Published:** 2024-01-02

**Authors:** Ebenezer Kwesi Armah-Ansah, Benedicta Bawa, Emmy Kageha Igonya

**Affiliations:** 1 Department of Population and Health, University of Cape Coast, Cape Coast, Ghana; 2 Population Dynamics Sexual and Reproductive Health Unit, African Population and Health Research Center, Nairobi, Kenya; 3 Department of Population and Development, National Research University - Higher School of Economics, Moscow, Russia; 4 One Trust LTD, East Legon, Accra, Ghana

**Keywords:** Guinea, Public health, Intention, SSA, Prevalence, Contraceptives

## Abstract

**Background:**

Contraceptive use is a key indicator of improving the health and well-being of women, mothers and their families, preventing unwanted pregnancies, and reducing maternal and child mortalities. Despite a lot of investments from the Government of Guinea to improve contraceptive use, studies reveal that contraceptive use still remains low in Guinea. However, the intention to use contraceptives in Guinea has not been well examined. Therefore, this study seeks to examine the factors associated with the intention to use contraceptives among women of reproductive age in Guinea.

**Methods:**

The study made use of data from the Guinea Demographic and Health Survey (GNDHS) conducted in 2018. For this study, we included a weighted sample of 6,948 women who were either married or cohabiting and responded to all the variables of interest. The data were analyzed using Stata version 14.2. Descriptive and multilevel logistic regression were carried out to examine the factors associated with the intention to use contraceptives. The results of multilevel logistic regression were presented using adjusted odds ratios at 95% confidence intervals and p-value < 0.05 to determine the significant associations.

**Results:**

The prevalence of intention-to-use contraceptives among women was 19.8% (95% CI18.3%–21.5%). Women with secondary/higher educational levels [aOR = 1.58, 95% CI = 1.26–1.99], women whose partners had secondary/higher educational level [aOR = 1.26, 95% CI = 1.04–1.52], women who were cohabiting [aOR = 1.74, 95% CI = 1.13–2.68] and were exposed to mass media [aOR = 1.60, 95% CI = 1.35–1.89] were likely to have higher intentions to use contraceptives. Additionally, women from the Kankan Region [aOR = 4.26, 95% CI = 2.77–6.54] and women who belong to the richer wealth quintile [aOR = 1.36, 95% CI = 0.91–1.89] were likely to have higher odds of intentions to use contraceptives. However, women aged 45–49 years, those from the Peulh ethnic group, and those who lack the competence to make healthcare decisions alone had lower odds of intention to use contraceptives.

**Conclusion:**

The study revealed a low prevalence of intention to use contraceptives among women of reproductive age in Guinea. The study has highlighted that both individual-level and household/community-level factors were significantly associated with the intention to use contraceptives. Therefore, policymakers and stakeholders need to consider these factors discussed in this paper when developing policies and interventions to promote and enhance intention-to-use contraceptives among women of reproductive age in Guinea. The findings call on the Government of Guinea and all stakeholders in Guinea to ensure that female education is promoted to help improve their social status, decision-making on fertility, and reduce fertility rates and maternal mortality.

## Background

Population growth is a major concern in many low-and-middle-income countries (LMICs), especially in sub-Saharan Africa (SSA) [1]. The population of SSA is estimated to increase more than expected in the next three decades, and it is projected to account for more than half of the world’s total population [1–4]. On average, SSA countries have fertility rates exceeding five children per woman [2]. It has been demonstrated that using contraceptives is an effective medical method for controlling fertility and enhancing mother-and-child health. [5].

The beginning of the 21st century saw a remarkable increase in the use of contraceptives in all parts of the world, including SSA [5]. The availability, accessibility, and utilization of contraceptives have contributed to more freedom independence and promoted gender equality through the sexual and reproductive health and rights of women [6]. Contraceptive methods, including pills, intrauterine devices, and condoms, which account for more than three-quarters of global contraceptive use, allow couples to have sex without any fear of pregnancies, sexually transmitted infections (STIs), or having to opt for abortion [6, 7].

Contraceptive use is a key indicator of increasing female education, women’s empowerment, improving the health and well-being of mothers and their families, and reducing maternal and child deaths [2, 8, 9]. Scholarly information has revealed that the intention to use contraceptives is a measure for women to better visualize their fertility needs and is more likely to translate to actual practice [10].

There have been a lot of investments and funding from local governments, community-based organizations, and extraterritorial organizations including non-governmental agencies and charity organizations, to improve contraceptive use in the last two decades [11, 12]. Specifically, the Government of Guinea has enacted a law called “The Reproductive Health” that details strategies that stakeholders can adopt to improve health standards and procedures for reproduction, the plan for securing reproductive health products, and the plan for repositioning family planning [13]. The government has received funding the from United Nations Population Fund (UNFPA), the United States Agency for International Development (USAID), the International Parenthood Federation (IPPF), and other donor partners to help execute intentions to use contraceptives and contraceptive-related initiatives and interventions [13].

The government has also created family planning access points at health facilities, established a budgeted 2019-2023 action plan, and created a 2015–2019 Strategic Plan for Health and Development of Adolescents and Youth that seeks to improve contraceptive use. These programs advocate for free family planning services for young people [14]. Despite these interventions, studies have revealed that contraceptive use still remains low in Guinea [15, 16].

Intention to use contraceptives among women of reproductive age is one of the key pathways to monitor the progress of Sustainable Development Goals three and five (SDGs 3 & 5) [17]. This supports universal access to sexual and reproductive healthcare services, including family planning, and achieving gender equality and empowering all women and girls by 2030 [18, 19].

One of the most effective ways to limit family size and unwanted pregnancies has been the intention to use contraceptives [20]. Although the intention to use contraceptives is recognised to be beneficial to the health of the mother and child, the family, and society [21], fertility is still high in Guinea [13]. The fertility rate in Guinea has not witnessed a significant decline. That is, the fertility rate of Guinea in 2005 has decreased from 5.7 births per woman of reproductive age to 4.8 births per woman of reproductive age in 2018 [13].

High fertility is a persistent problem for women of reproductive age, stakeholders, governments, and their agencies [20]. Empirical studies have shown that intention to use contraceptives among women of reproductive age in SSA has been affected by their partners’ preferences for fertility and other sociodemographic factors like age, educational level, exposure to mass media, marital status, place of residence, the number of children, distance to a health centre, the ideal number of children, employment status, and religion [20–23].

Studies on contraceptives in Guinea have focused on adolescents and young women [15, 16, 24, 25] and married women [26]. However, there is a paucity of studies focusing on the multilevel prevalence and factors associated with the intention to use contraceptives among women of reproductive age in Guinea. The multilevel factor approach will help to improve our understanding of both individual and household/community level factors that are statistically significant to the intention to use contraceptives among women of reproductive age in Guinea. Hence, this research seeks to fill the gap by examining the prevalence and factors associated with the intention to use contraceptives among women of reproductive age in Guinea. Therefore, this current study will provide up-to-date evidence for policymakers and other program managers to design and implement programs that may be appropriate interventions to increase the intention of contraceptive use among women of reproductive age in Guinea.

## Materials and methods

### Data source and study population

The study made use of data from the most recent Guinea Demographic and Health Survey (GNDHS), conducted in 2018. The DHS is a countrywide representative survey undertaken over a five-year period in several LMICs in Asia and Africa [27]. It focuses on demographic and health issues by interviewing women in their reproductive years (15–49 years). The DHS follows standardized procedures in areas such as sampling, questionnaires, data collection, cleaning, coding, and analyses, which allow for comparison across countries. Details of the methodology, instruments, pretesting of the instruments, training, and recruitment of enumerators are documented in the final report of the 2018 GNDHS [13]. The dataset is freely available for download at: https://dhsprogram.com/data/dataset/Guinea_Standard-DHS_2018.cfm?flag=1. We relied on the “Strengthening the Reporting of Observational Studies in Epidemiology” (STROBE) statement in writing the manuscript [28].

### Study population and inclusion criteria

**Fig. 1:**
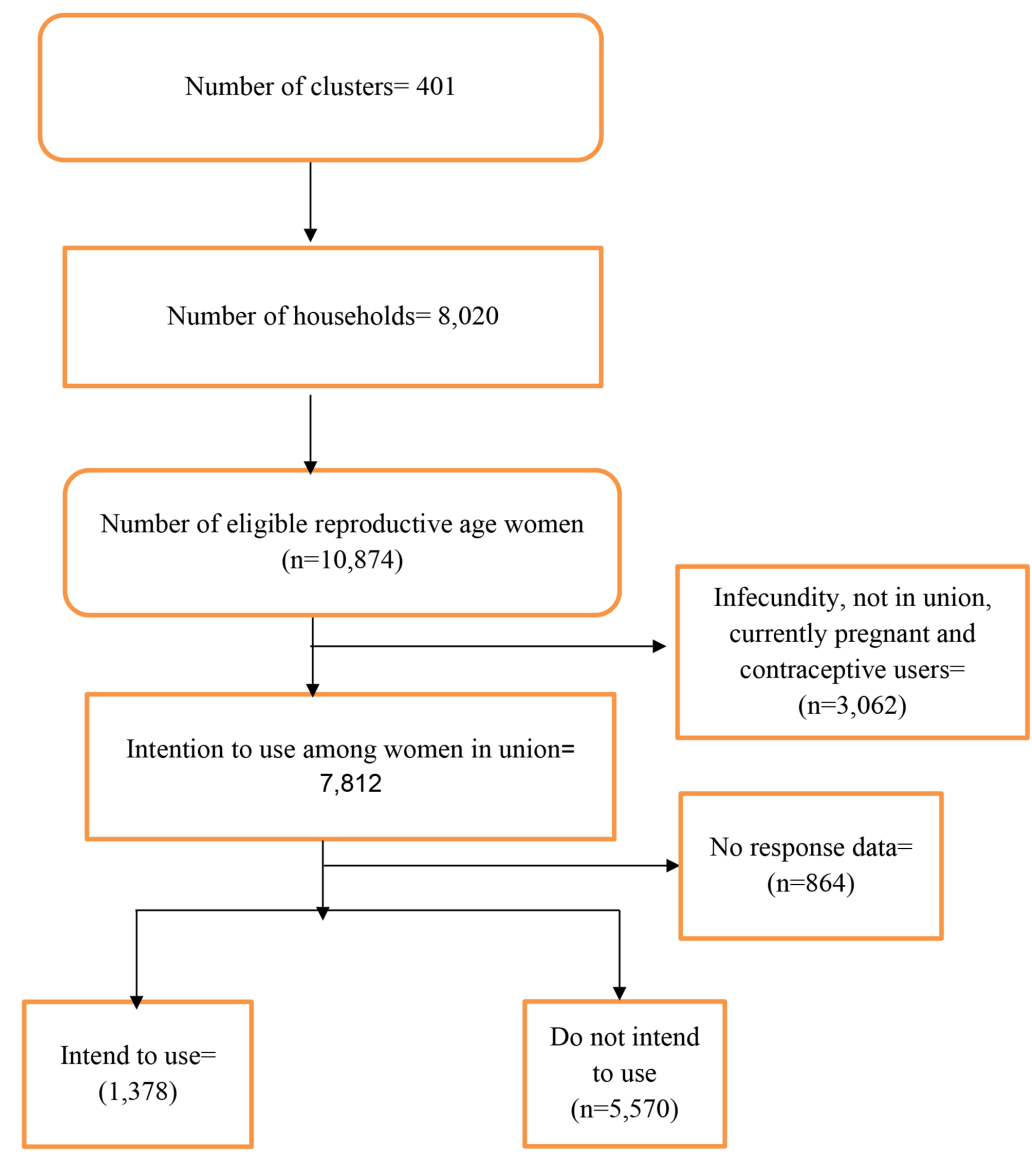
Sampling procedure

For this study, we used the women’s recode file with a weighted sample of 6,948 reproductive-age women who were either cohabiting or married and had complete cases on the variable of interests [Fig. 1]. The study excluded women with infecundity, who are currently contraceptive users, and who are currently pregnant [3, 21].

### Description of variables

#### Outcome variables

The outcome variable for the study was the intention to use contraceptives among reproductive-age women who are either cohabiting or married. This variable has three responses: “use later,” “unsure about use,” and “does not intend to use.” For this study, the response categories were recoded as ”do not intend to use” = “no,” which included do not intend to use and unsure about use, while ”intend to use” = “yes” was intend to use. Other DHS studies employed similar coding [3, 22, 29].

#### Independent variables

Based on the literature, sixteen independent variables were investigated as factors associated with the intention to use contraceptives among women of reproductive age. These variables were considered based on literature [3, 30–32]. The variables were grouped as individual and household/community-level factors. The individual-level variables included age (15–19, 20–24, 25–29, 30–34, 35–39, 40–44, 45–49); education (no education, primary education, secondary/higher); partner education (no education, primary school, secondary school/higher); marital status (married, cohabiting); employment status (not working, working). Additionally, parity (no birth, one birth, two births, three births, four or more births), mass media (no, yes), age at first sex (< 20, $\ge 20$) and religion (Muslim, Christian, Traditional, no/other religion).

The household/community-level factors are healthcare decision-making capacity (alone, not alone); ethnicity (Soussou, Peulh, Malinké, Forestier/others); region (Boke, Conakry, Faranah, Kankan, Kindia, Labe, Mamou, N’zerekore); wealth (poorest, poorer, middle, richer, richest); place of residence (urban, rural); community literacy level (low, medium, high); and community socioeconomic status (low, moderate, high). The community literacy level is measured as the proportion of women who completed at least primary education, and community socioeconomic variables were obtained by aggregating the individual-level variables into clusters.

### Statistical analysis

The data were processed and analyzed using Stata version 14.2. First, a descriptive analysis was used to describe the prevalence of the intention to use contraceptives. This was followed by a bivariate result on the distribution of the independent variables against the outcome variable using chi-square [χ2] test of independence. Variables that showed statistical significance at a p-value of 0.05 were moved into the multilevel logistic regression analysis. Therefore, women were nested within clusters and considered as random effects to cater for the unexplained variability at the household/community level [9, 32]. Using the variance inflation factor (VIF), the multicollinearity test showed that there was no evidence of collinearity among the explanatory variables (mean VIF = 2.02).

In the multilevel logistic regression, which has fixed and random effects, there are 4 models. The first model (Model 0) was an empty model where no explanatory variable was used and the result showed the variance of intention to use contraceptives attributable to the distribution of the primary sampling units. Model 1 took into account only individual -level variables, while Model 2 had only household/community-level variables. Model 3, which was the complete model, had both the individual and household/community-level variables. The results were presented as adjusted odds ratios (aORs) with their corresponding 95% confidence intervals, signifying their level of precision. The STATA command ‘melogit’ was used in fitting these models. Model comparison was done using the log-likelihood ratio (LLR) and Akaike’s Information Criterion (AIC). The complex nature of the sampling structure of the data was adjusted using the Stata Survey command ‘svyset v021 [pweight = wt], strata (v023)’.

## Results

### Prevalence

**Fig. 2:**
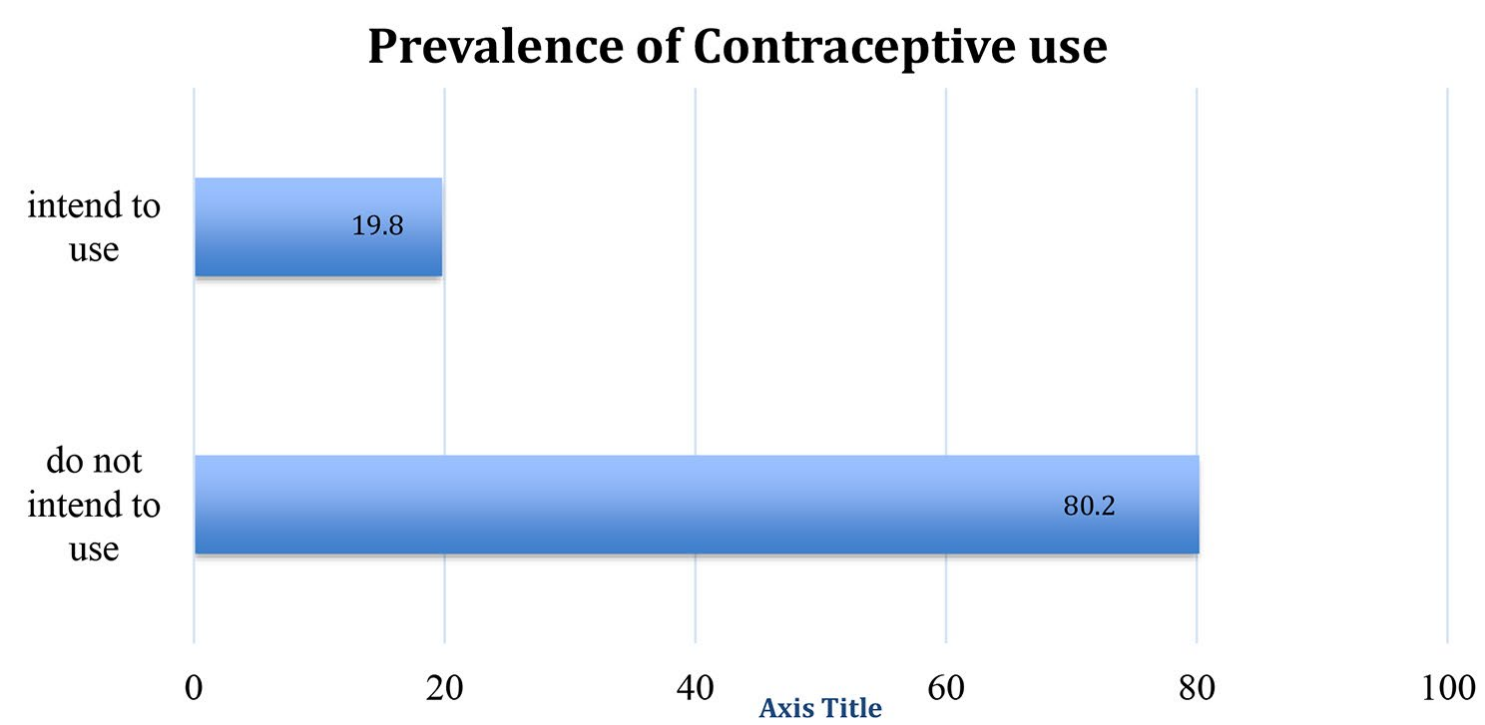
Prevalence of intention to use contraceptives, 2018 GNDHS

From the study, the prevalence of intention to use contraceptives among women of reproductive age is 19.8% (95% CI:0.18–0.21) (see Fig. 2).

### Socio-demographic characteristics of women of reproductive age

The results (Table 1) showed that 21.2% of the respondents were aged 25–29; 81.0% had no formal education; 74.2% of respondents’ partners had no formal education; and 97.8% of the participants were married. More than two-thirds (74.9%) were working, 43.5% had four or more births, 63.2% indicated that they had access to mass media, and 89.1% were Muslims. More than three-quarters (88.1%) of the respondents had their first sex before age 20 years; 41.2% were Peulh; and 15.2% reside in Kindia Region. With regard to healthcare decision-making capacity, 90% indicated that they do not make decisions alone; 23.7% were in the poorest wealth quintile 72.1% resided in rural areas; 40.5% and 65.8% had low community literacy levels and low community socioeconomic status, respectively.

**Table 1:** Socio-demographic characteristics of women of reproductive age (Weighted, N = 6,948)

Variables	Weighted N (%)	Intention to use contraceptive (%)	χ2, p-value
***Individual level factors***			
**Age**			172.0, **< 0.001**
15–19	654 (9.4)	27.9	
20–24	1,026 (14.8)	24.2	
25–29	1,477 (21.2)	24.3	
30–34	1,138 (16.4)	22.1	
35–39	1,074 (15.5)	16.9	
40–44	807 (11.6)	12.5	
45–49	772 (11.1)	7.1	
**Education**			134.9, **< 0.001**
No education	5,630 (81.0)	17.3	
Primary	629 (9.1)	27.3	
Secondary+	689 (9.9)	33.7	
**Partner’s Education**			78.4, **< 0.001**
No education	5,158 (74.2)	17.4	
Primary	483 (7.0)	22.9	
Secondary+	1,307 (18.8)	28.2	
**Marital status**			18.0, **< 0.001**
Married	6,797 (97.8)	19.4	
Cohabitation	151 (2.2)	38.7	
**Employment status**			0.3, 0.57
Not working	1,744 (25.1)	18.8	
working	5,204 (74.9)	20.2	
**Parity**			6.0, 0.20
None	667 (9.7)	19.8	
One birth	1,009 (14.5)	22.0	
Two births	1,107 (15.9)	19.2	
Three births	1,141 (16.4)	21.0	
Four or more births	3,024 (43.5)	18.9	
**Mass media**			165.1, **< 0.001**
No	2,554 (36.8)	12.1	
Yes	4,394 (63.2)	24.4	
**Religion**			3.5, 0.317
Muslim	6,191 (89.1)	19.5	
Christian	670 (9.6)	21.9	
Traditional	3 (0.1)	0.0	
No/other Religion	84 (1.2)	30.8	
**Age at first sex**			7.1, **0.008**
< 20	6,123 (88.1)	20.3	
$\ge 20$	825 (11.9)	16.1	
**Healthcare Decision Making Capacity**			11.2, **0.001**
Alone	693 (10.0)	25.7	
Not Alone	6,255 (90.0)	19.2	
**Ethnicity**			120.9, **< 0.001**
Soussou	1,270 (18.3)	20.2	
Peulh	2,861 (41.2)	13.8	
Malinké	1,968 (28.3)	26.6	
Forestier/Others	849 (12.2)	24.0	
**Region**			255.0, **< 0.001**
Boke	794 (11.4)	12.3	
Conakry	817 (11.8)	21.4	
Faranah	715 (10.3)	31.1	
Kankan	971 (14.0)	34.2	
Kindia	1,055 (15.2)	14.3	
Labe	849 (12.2)	15.1	
Mamou	718 (10.3)	15.1	
N’zerekore	1,029 (14.8)	16.0	
**Wealth**			74.9, **< 0.001**
Poorest	1,644 (23.7)	15.5	
Poorer	1,549 (22.3)	18.8	
Middle	1,438 (20.7)	17.0	
Richer	1,269 (18.3)	25.7	
Richest	1,048 (15.0)	25.0	
**Place of residence**			47.4, < **0.001**
Urban	1,938 (27.9)	25.0	
Rural	5,010 (72.1)	17.9	
**Community literacy level**			31.8, **< 0.001**
Low	2,814 (40.5)	18.3	
Medium	2,474 (35.6)	18.6	
High	1,660 (23.9)	24.2	
**Community socioeconomic status**			54.8, **< 0.001**
Low	4,571 (65.8)	17.3	
Moderate	672 (9.7)	25.8	
High	1,705 (24.5)	24.4	

The result shows the highest prevalence of intention to use contraceptives among women aged 15–19 (27.9%), those with secondary/higher education (33.7%), partners with secondary/higher education (28.2%), cohabiting women (38.7%), and working women (20.2%). Women with one birth (22.0%), women who had exposure to mass media (24.4%), and women who make healthcare decisions alone (25.7%) had the highest prevalence of the intention to use contraceptives. With regards to religion, and age at first sex, the highest prevalence was found among Christians (21.9%) and women who had sex before the age of 20 (20.3%), respectively. The highest prevalence of intention to use contraceptive was found among women who were in the richest wealth quintile (25.0%), women residing in an urban setting (25.0%), women affiliated with the Malinké ethnic group (26.6%), women who reside in Kankan Region (34.2%), women in high community literacy level (24.2%), and women with moderate community socioeconomic status (25.8%).

### Multilevel logistic regression analysis of intention to use contraceptives

In Table 2, Model 3 presents the results of the multilevel logistic analysis on the factors associated with the intention to use contraceptives among women of reproductive age. With regards to age, women aged 45–49 years had lower odds of intention to use contraceptives [aOR = 0.23, 95% CI = 0.17–0.33] compared to women aged 15–19 years. Women with secondary/higher educational levels [aOR = 1.58, 95% CI = 1.26–1.99] had higher odds of intention to use contraceptives compared with women with no education. Partners with secondary/higher education had a greater likelihood of contraceptive use [aOR = 1.26, 95% CI = 1.04–1.52] than those with no education. Women who were cohabiting had higher intention to use contraceptives [aOR = 1.74, 95% CI = 1.13–2.68] than married women. It was found that women who were exposed to mass media were likely to have higher intention to use contraceptives [aOR = 1.60, 95% CI = 1.35–1.89].

**Table 2:** Multilevel logistic regression analysis of intention to use contraceptives

Variables	Model 0	Model 1 aOR (95% CI)	Model 2 aOR (95% CI)	Model 3 aOR (95% CI)
***Fixed effect***				
***Individual level factors***				
**Age**				
15–19		Ref		Ref
20–24		0.85 (0.66–1.09)		0.84 (0.65–1.08)
25–29		0.94 (0.74–1.19)		0.95 (0.75–1.21)
30–34		0.87 (0.67–1.12)		0.86 (0.67–1.12)
35–39		0.65** (0.49–0.84)		0.65** (0.50–0.85)
40–44		0.45*** (0.33–0.60)		0.44*** (0.32–0.59)
45–49		0.24*** (0.17–0.33)		0.23*** (0.17–0.33)
**Education**				
No education		Ref		Ref
Primary		1.42** (1.14–1.76)		1.44 (1.16–1.79)
Secondary+		1.63*** (1.30–2.04)		1.58*** (1.26–1.99)
**Partner’s Education**				
No education		Ref		Ref
Primary		1.05 (0.81–1.36)		1.04 (0.80–1.35)
Secondary+		1.33** (1.00-1.59)		1.26* (1.04–1.52)
**Marital status**				
Married		Ref		Ref
Cohabitation		1.81* (1.18–2.77)		1.74* (1.13–2.68)
**Age at first sex**				
< 20		Ref		Ref
$\ge 20$		0.85 (0.68–1.06)		0.89 (0.71–1.11)
**Mass media**				
No		Ref		Ref
Yes		1.74*** (1.47–2.05)		1.60*** (1.35–1.89)
***Household and Community level factors***				
**Healthcare Decision Making Capacity**				
Alone			Ref	Ref
Not Alone			0.79* (0.64–0.98)	0.69** (0.55–0.86)
**Ethnicity**				
Soussou			Ref	Ref
Peulh			0.45*** (0.35–0.59)	0.49*** (0.37–0.64)
Malinké			0.63**(0.47–0.85)	0.66**(0.49–0.88)
Forestier/Others			1.10 (0.76–1.59)	1.00 (0.69–1.46)
**Region**				
Boke			Ref	Ref
Conakry			0.92 (0.59–1.43)	0.91 (0.59–1.41)
Faranah			3.91***(2.59–5.90)	3.75***(2.50–5.63)
Kankan			4.69*** (3.05–7.22)	4.26***(2.77–6.54)
Kindia			0.90 (0.61–1.33)	0.94 (0.64–1.37)
Labe			2.39*** (1.56–3.65)	2.43*** (1.60–3.69)
Mamou			2.25*** (1.48–3.42)	2.72*** (1.80–4.12)
N’zerekore			1.01 (0.63–1.62)	1.05 (0.65–1.69)
**Wealth**				
Poorest			Ref	Ref
Poorer			1.12 (0.91–1.38)	1.07 (0.87–1.33)
Middle			0.84 (0.67–1.05)	0.81 (0.64–1.02)
Richer			1.56** (1.18–2.06)	1.36* (0.91–1.89)
Richest			1.76** (1.24–2.49)	1.31 (0.91–1.89)
**Place of residence**				
Urban			Ref	Ref
Rural			0.89 (0.56–1.41)	0.90 (0.57–1.41)
**Community literacy level**				
Low			Ref	Ref
Medium			1.08 (0.83–1.39)	1.00 (0.78–1.29)
High			1.39 (0.89–2.16)	1.20 (0.78–1.85)
**Community socioeconomic status**				
Low			Ref	Ref
Moderate			0.98 (0.66–1.45)	0.95 (0.65–1.40)
High			1.06 (0.61–1.82)	0.98 (0.57–1.66)
**Random effect results**				
PSU variance (95% CI)	0.94 (0.74–1.19)	0.74 (0.57–0.96)	0.49 (0.36–0.66)	0.43 (0.31–0.59)
ICC	0.22	0.18	0.13	0.11
LR Test	X^2^ = 385.81, p < 0.001	X^2^ = 259.91, p < 0.001	X^2^ = 141.59, p < 0.001	X^2^ = 109.72., p < 0.001
Wald χ2	Ref	253.68	214.30	419.41
Model fitness				
Log-likelihood	-3248.11	-3106.26	-3148.98	-3025.81
AIC	6500.21	6242.53	6341.96	6121.63
BIC	6513.90	6345.22	6492.57	6361.24
N	6,948	6,948	6,948	6,948

*Source: 2018 GNDHS*

^*^p < 0.05, ^**^p < 0.01, ^***^p < 0.001,

*Ref-Reference category; PSU Primary Sampling Unit; ICC Intra-Class Correlation; aOR adjusted odds ratio; LR Test Likelihood Ratio Test; AIC Akaike’s Information Criterion; BIC Bayesian Information Criterion*

With regards to healthcare decision-making capacity, intention to use contraceptives was lower among women who do not have the capacity to make healthcare decisions alone [aOR = 0.69, 95% CI = 0.55–0.86] compared to those who have the capacity to make healthcare decisions alone. Women affiliated with the Peulh ethnic group had lower odds of intention to use contraceptives [aOR = 0.49, 95% CI = 0.37–0.64] compared to those affiliated with Soussou ethnic groups. Women who live in the Kankan Region had higher odds of intention to use contraceptives [aOR = 4.26, 95% CI = 2.77–6.54] compared to those who live in the Boke Region. Regarding wealth quintiles, women in the richer wealth quintile had higher odds of intention to use contraceptives [aOR = 1.36, 95% CI = 0.91–1.89] than their counterparts who are in the poorest wealth quintile (See Table 2).

### Random effects (measures of variation) results

The result of the random effect (Table 2) indicates that there was a statistically significant variation in the intention to use contraceptives across the clusters. In the empty model, there were substantial variations in the likelihood of intention to use contraceptives across the clustering of the PSUs [σ2 = 0.94, 95% CI 0.74–1.19]. The ICC value for Model 0 shows that 22% of the variation in the intention to use contraceptives was attributed to the between-cluster variations of the characteristics. The variation between clusters then decreased to 18% in Model 1, which was the individual-level only model. The ICC further decreased to 13% in Model 2, which had only household/community-level factors model. In the final model (Model 3), the between-cluster variation further decreased to 11%. This can be attributed to the differences in the clustering of the PSUs, which account for the variations in the intention to use contraceptives. From the model specification analysis, Model 3, which is the complete model with individual-level and household/community-level factors, had the lowest AIC compared to the other models, affirming the goodness of the model (see Table 2).

## Discussion

This study examined the prevalence and factors associated with intention to use contraceptives among women of reproductive age in Guinea, a country in West Africa that is noted to have a history of low contraceptive use and high maternal and child mortalities [15, 33]. The study revealed that two out of ten women in Guinea had the intention to use contraceptives (19.8%). The study results further indicated that women’s age, educational level, partners educational level, marital status, mass media, healthcare decision-making capacity, ethnicity, region, and wealth were significantly associated with the intention to use contraceptives among women of reproductive age in Guinea.

The prevalence found in this present study is higher than a prevalence of 18.2% in western Ethiopia [34]. However, it is lower than those reported by studies conducted in Mozambique [22], southern Ethiopia [35], and SSA [3]. The low prevalence of the intention to use contraceptives demonstrates that the use of contraceptives to regulate fertility and improve mother and child health may still be a significant problem in Guinea. The difference in prevalence could be due to variation in the study design, access to information and services, myths, fear of side effects, socio-economic status, and community norms and factors [34–36].

In this study, women aged 45–49 had lower odds of intention to use contraceptives in Guinea. This finding is consistent with other studies conducted in Ethiopia [18], Nigeria [28], Malawi [29], and SSA [3]. However, studies conducted in the north and northwest of Ethiopia [37, 38] were contrary to the finding of this study. The plausible reason for our finding could be that older women may have experienced a decline in coital frequency. Additionally, these older women may be unwilling and uncomfortable discussing their reliance on other conventional methods, including string ties. This could have resulted in a lower intention to use contraceptives [3, 29].

The study found a significant association between the educational level of respondents and their intention to use contraceptives. Compared to women with no formal education, women with secondary/higher educational levels were found to have higher odds of having the intention to use contraceptives. The finding resonates with other studies conducted in SSA [3, 29, 35, 37, 39]. However, this finding is inconsistent with other studies done in Ethiopia [17, 40]. This could be attributed to the fact that educated women had access to different sources of information on contraceptives and were empowered. This will not only increase women’s social status but also their decision-making on fertility issues, and reduce fertility rates, and maternal mortality [21, 41].

The education of the respondent’s partner was one of the key factors associated with the intention to use contraceptives. This investigation is consistent with other studies conducted in northern Ethiopia [17], Cameroon [42], and Nigeria [43]. However, another study in Ethiopia [39] revealed that the education of the respondent’s partner and their intention to use contraceptives were not significant. The possible explanation could be that an educated partner may motivate and support their wife’s intention to use contraceptives since they may be well informed about the importance of contraceptives.

The likelihood of an intention to use contraceptives among women cohabiting was higher than that of women who were married. The finding of this study is in agreement with a previous study across the SSA [3]. This evidence suggests that cohabiting women may choose to delay having children because of the sociocultural norms that most SSA cultures have about births outside of marriage [44].

Consistent with previous studies in SSA [3], Uganda [45], West Africa [46], and Pakistan [47], this study reveals that women who were exposed to mass media were more likely to have intentions to use contraceptives. This is an indication that women trust the various mass media channels and, hence receive the health benefits of contraceptive use. That is, mass media, including radio, television, and newspapers, have been channels for promoting desirable lifestyles, knowledge, healthy behavior, and attitudes towards contraceptives [47].

Furthermore, women who did not make healthcare decisions alone reported a lower likelihood of intending to use contraceptives. The finding contradicts other studies in Ethiopia [10, 34], Mozambique [22], and Pakistan [47]. This result shows the pivotal role that partners or family members play in influencing the intention of women to use contraceptives in Guinea. Given the importance of healthcare decision-making capacity, the promotion of and education about contraceptives should not only focus on women but should include their partners and immediate family members. This will help women gain autonomy in their healthcare decision-making capacity.

In agreement with previous studies done in SSA [16, 30], this study found that ethnicity is significantly associated with the intention to use contraceptives. Sidibé et al., [16] similarly found that women of reproductive age who belong to the Peulh ethnic group had a lower likelihood of intention to use contraceptives in Guinea. In explaining the plausible reason accounting for this observation, Ahinkorah et al., [48] contended that women of reproductive age belonging to the Peulh ethnic group had little or no support from their partners and may be restricted by their religious beliefs.

Another important factor that significantly influenced the intention of contraceptive use in this study was region. The odds of an intention to use contraceptives were higher among those women living in the Kankan region. This finding is similar to studies conducted in Guinea [16, 24]. This might be due to residual confounding, as data have shown that women from the Conakry region have the highest level of education, access to the contraceptives, and high health service coverage [49]. This finding calls for further studies to understand the reasons for higher odds of intention to use contraceptives in the Kankan region.

Finally, we found the wealth quintile to be significantly associated with the intention to use contraceptives among women of reproductive age in Guinea. The odds of an intention to use contraceptives were higher among women in the richer and richest wealth quintiles. This finding is supported by studies done in Guinea [16], SSA [21], Mozambique [50], Ethiopia [51], and Ghana [52]. It is possible that women who belong to the richer and richest wealth quintiles could pay for the costs associated with contraceptive uptake [53].

### Strengths and limitations

This study used the most current nationally representative data from the Guinea Demographic and Health Survey to examine the factors associated with the intention to use contraceptives. Additionally, the authors estimated the cluster effect on intention to use contraceptives among women of reproductive age using a mixed-effects analysis, an appropriate statistical approach. The methods employed in sampling and data collection also support the representativeness of the study. Thus, the findings and recommendations can be applied to all women of reproductive age in Guinea. Also, the cross-sectional nature of this study does not allow for causality to be inferred from the findings. Furthermore, the data were collected retrospectively which may have a recall bias and could lead to over-or under-reporting.

### Conclusions and recommendations

The study revealed a low prevalence of intention to use contraceptives among women of reproductive age in Guinea. The study has highlighted that both individual-level and household/community-level factors were significantly associated with the intention to use contraceptives. Therefore, policymakers and stakeholders need to consider these factors when developing policies and interventions to promote and enhance intention-to-use contraceptives among women of reproductive age in Guinea. The findings call on the Government of Guinea and all stakeholders in Guinea to ensure that female education is promoted. This could help improve their social status, decision-making on fertility and reduce fertility rates, and maternal mortality. To improve the intention to use contraceptives among women in Guinea, health education programs through the various channels of mass media should focus on women of all ages and Peulh ethnic group. These findings also support a call for contraceptive strategies that target the involvement of their partners. This is an essential tool that ensures that women have sexual autonomy.

## Data Availability

Data is available on https://dhsprogram.com/data/dataset/Guinea_Standard-DHS_2018.cfm?flag=1. Other authors would be able to access or request these data in the same manner as we did from DHS program. Authors did not have any special access or request privileges that others would not have.

## Abbreviations

LMICs: Low-and-middle-income countries
SSA: Sub-Saharan Africa
STIs: Sexual Transmitted Infections
GNDHS: Guinea Demographic and Health Survey
aOR: Adjusted odds ratio
CI: Confidence intervals
